# Long term outcome and elasticity of a polyester mesh used for laparoscopic ventral hernia repair

**DOI:** 10.1007/s10029-021-02486-6

**Published:** 2021-08-23

**Authors:** P. J. O’Dwyer, C. Chew, H. Qandeel

**Affiliations:** 1 School of Medicine, Dentistry and Medicine, University of Glasgow, Glasgow, UK; 2 Department of Radiology, University Hospital Hairmyres, Glasgow, UK; 3 Department of Surgery, Hashemite University, Zarqa, Jordan

**Keywords:** Hernia, Laparoscopic, Mesh, Computed tomography, Late recurrence

## Abstract

**Background:**

Repair of a ventral hernia is increasingly being performed by a laparoscopic approach despite lack of good long term follow up data on outcomes. The aim of this study was to examine the long term performance of a polyester mesh and to assess its elastic properties in patients undergoing laparoscopic ventral hernia repair.

**Methods:**

All patients being assessed for a ventral hernia repair between August 2011 and November 2013 were placed on a prospective database. Those undergoing laparoscopic repair with a polyester mesh were seen at clinic at one month and one year, while their electronic records were assessed at 34 months (range 24–48 months) and 104 months (range 92–116 months). In addition, CT scans of the abdomen and pelvis performed for any reason on these patients during the follow up period were reviewed by a consultant gastrointestinal radiologist. Mechanical failure testing of the mesh was also performed.

**Results:**

Thirty-two of the 100 patients assessed for ventral hernia repair had a laparoscopic repair with a polyester mesh. Nineteen (59%) had CT scans performed during the follow-up period. No recurrence was recorded at 34 months, while three (9.4%) had a recurrence at 104 months. Two had central breakdown of the mesh at 81 and 90 months, while 1 presented acutely at 116 months after operation. Mesh had stretched across the defect by an average of 21% (range 5.7–40%) in nine patients. Mechanical testing showed that this mesh lost its elasticity at low forces ranging between 1.8 and 3.2 N/cm.

**Conclusion:**

This study shows that late recurrence is a problem following laparoscopic ventral hernia repair with polyester mesh. The mesh loses it elasticity at a low force. This combined with degradation of mesh seems the most likely cause of failure. This is unlikely to be a unique problem of polyester mesh and further long-term studies are required to better assess this operative approach to ventral hernia repair.

## Introduction

Repair of a ventral hernia is a common operation that is increasingly being undertaken by a laparoscopic approach [1]. The advantages of the laparoscopic over the open approach include shorter hospital stay, a more rapid return to normal activities and perhaps most importantly less wound complications. In a recent nationwide study of over 5000 patients recurrence rates were similar for open and laparoscopic repair after an average follow-up of 4 years [1]. This study also indicated that for defects between 2 and 6 cm, the laparoscopic approach may be superior to open repair.

Most meshes are sufficiently strong to resist the pressures exerted on them by increased intraabdominal pressure such as with coughing. However, meshes degrade over time and lose their elasticity. A recent study has shown that a lightweight polyester mesh ruptured centrally when used to augment open ventral hernia repair [2]. A meta-analysis and systematic review of over 10,000 patients comparing polyester with polypropylene meshes, however, found no difference in recurrence rates between the products [3]. Follow-up in most of these studies was short with little data available on outcomes beyond 5 years.

The aim of this study was to examine the long-term performance of a polyester mesh and to assess its elastic properties in patients undergoing laparoscopic ventral hernia repair.

## Methods

Between august 2011 and November 2013 all patients with a ventral hernia under the care of one surgeon were maintained on a prospective database. Data recorded included age, gender, Body mass index (BMI), American Society of Anaesthetist grade (ASA) type of hernia, defect width, clean or contaminated wounds, etc. Operative details including type of operation, mesh used, operative times and complications were also recorded, as were hospital stay and postoperative complications.

Patients considered suitable for a laparoscopic repair were those with a defect width of between 2 and 6 cm or those with high comorbidity irrespective of defect size. A monofilament polyester mesh with a hydrophilic porcine dermis collagen barrier (Parietex Composite Optimised Mesh, Covidien, New Haven, CT, USA) was used for all patients.

The abdominal cavity was accessed at Palmer’s point using an optical port (Endopath Xcel, Ethicon). Two additional 5-mm ports were placed under direct vision and used to reduce the hernia and take down any adhesions present. An intraperitoneal onlay mesh was then inserted and placed across the defect with at least a 5-cm overlap. The mesh was secured with two rows of tacks (ProTack Covidien New Haven, CT, USA) and four transfacial sutures. An additional optical port and a 5-mm working port was also inserted on the patient’s right side under direct vision. This allowed tacking from both sides and ensure the mesh was not lax following repair and overlap of defect was similar on both sides. All 10- to 12-mm port sites were closed with an absorbable suture.

Patients were followed-up at clinic at 1 month and 1 year while electronic notes were assessed at two further time points to access long-term outcomes. In addition, CT scans of the abdomen and pelvis performed for any reason during follow-up were reviewed by a consultant radiologist to determine if a recurrent hernia was present on CT. Also the mesh across the defect was measured on CT to determine if it had stretched during follow-up. Measurements were taken axial view where the defect was maximal and followed the expected normal contours of the abdominal wall. The mesh length was measured at the marked points of the defect edge on the same CT image.

In addition, polyester mesh (Parietex Composite) was subjected to failure testing by a Zwick-Roell Z2.0 machine (Zwick-Roell, Ulm, Germany). The mesh was cut into 140 mm by 25 mm specimens in the longitudinal (warp) and transverse (weft) direction. Specimens were hydrated in saline at 37 degrees Celsius for at least 10 min before testing. Mesh was placed between grips of the machine with 20 mm overlap on each side. The mesh was then subjected to loading at a rate of 10 mm per minute. These settings were chosen in accordance with the British Standards Institute. Each test was ended when the mesh ruptured.

### Statistics

Data were expressed as mean and standard deviation where appropriate. Parametric data was compared using a *T* test while nonparametric data were analysed using a Chi squared test. Analysis was performed using IBM Statistics for windows, version 22.0 (Armonk New York USA, IBM corp.)

## Results

Thirty-two of the 100 patients evaluated for ventral hernia repair were considered suitable for a laparoscopic approach. These patients were significantly older than their open counterparts but demographic data were otherwise similar (Table 1). The average defect width was 6.2 cm (range 2–12 cm) while the average mesh used to cover this defect was 17 cm (range 15–25 cm). There were no intraoperative complications with this group of patients while the average hospital stay was 2 days. Four patients had a seroma while one had severe pain postoperatively.

**Table 1:** Demographic details of patients undergoing laparoscopic and open ventral hernia repair

	Laparoscopic (*N* = 32)	Open (*N* = 68)	*p* value
Age years	63.5 (11.5)	53.7 (15.5)	0.001
Male/female	10/22	33/35	0.158
BMI	31.7 (4.9)	32.2 (6.9)	0.318
ASA score	2.39 (0.8)	2.21 (0.62)	0.158
Incisional hernia*	27	59	0.750

Data area expressed as mean (standard deviation) where appropriate

*Others included umbilical (5), epigastric (5), Spigelian (2) and port site hernia (2)

Electronic records of patients were reviewed at a mean follow-up of 34 months (range 24–48 months) and again at a mean follow-up of 104 months (range 92–116 months). Nineteen (59%) of the 32 patients operated on had an abdominopelvic CT on follow-up, the indications for CT are shown on Table 2. At 34 months no recurrences were recorded while at 104 months, three (9.4%) patients were noted to have a recurrence on CT scan. Two of these were noted on staging CT for cancer, while one presented acutely with incarcerated omentum in a hernia sac. The interval between operation and recurrence was 81, 90 and 116 months, respectively. Two of the recurrences involved central rupture of the mesh while the third was observed between two, tacks lateral to the original defect. An additional patient had a large pelvic cancer which ruptured through the mesh 47 months after her hernia repair.

**Table 2:** Indications for CT scan

Indication	Number
Cancer*	10
Pain	5
Sepsis	2
Obstruction^#^	1
Incarcerated hernia	1

*This included diagnosis, staging or follow-up of a patient with cancer

^#^This patient had adhesive obstruction which settled on conservative treatment

### Elasticity of mesh

Ten meshes were tested − 5 in the longitudinal direction and 5 in the transverse direction. Mesh lost its elasticity at a force of 1.8 N/cm in the longitudinal direction and 3.2 N/cm in the transverse direction. This was associated with an increase in length of 22 and 5 mm, respectively. Mesh ruptured at a force of 20.8 n/cm in the longitudinal direction and 42.1 N/cm in the transverse direction. The increase in length associated with this was 79 and 36 mm, respectively (Table 3).

**Table 3:** Mechanical properties of polyester mesh

Mesh direction	Loss of elasticity (N/cm)*	Increase in length (%)	Rupture (N/cm)*	Increase in length (%)
Longitudinal (SD)	1.8 (0.1)	22 (2)	20.8 (1.6)	79 (3)
Transverse (SD)	3.2 (0.2)	5 (0)	42.1 (3.6)	36 (3)

Data are expressed as mean (standard deviation)

^*^Force (N/cm): Newton per centimetre

**Fig. 1:**
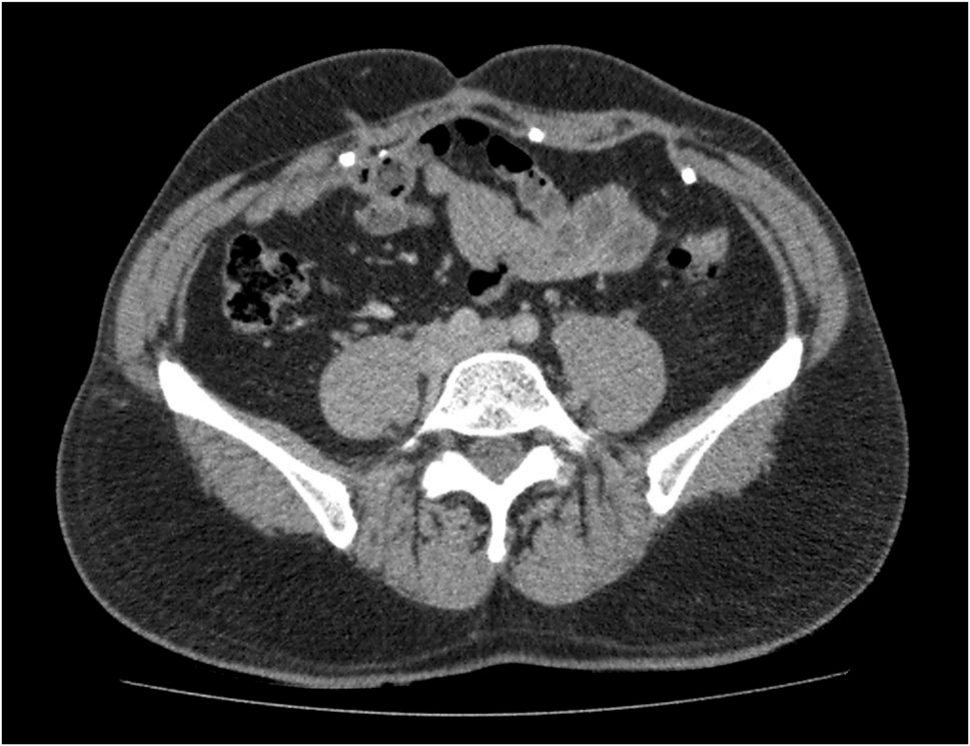
Mesh has stretched by 40% across a 2.5-cm defect on the patient’s left side 2 years after hernia repair. Note the mesh is also stretched across a midline defect but to a lesser degree

**Fig. 2:**
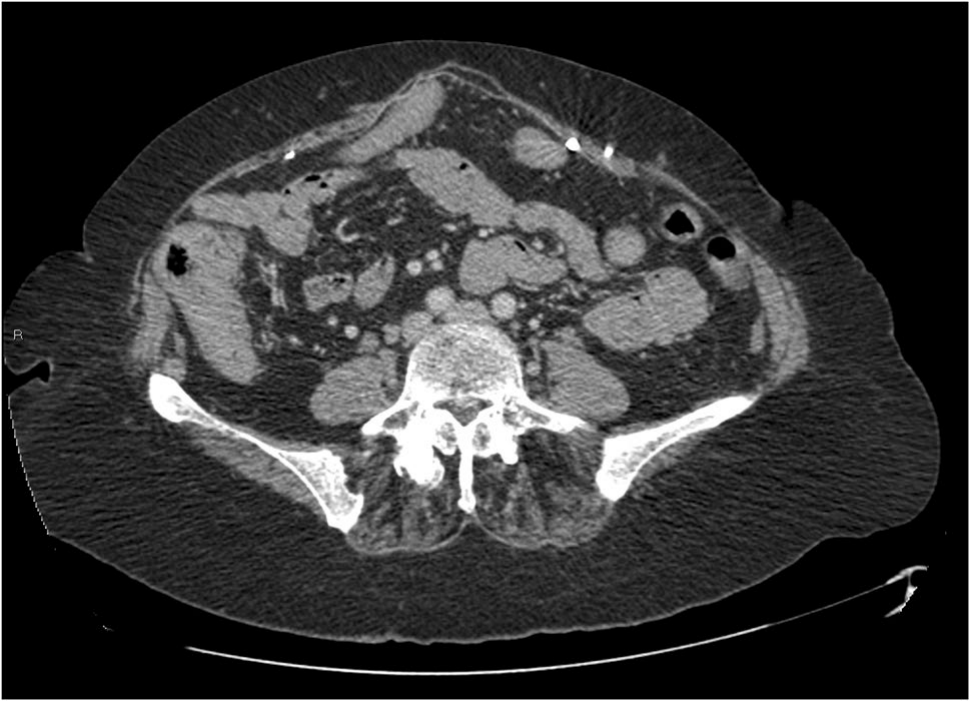
Mesh has stretched by 24% across a 6.5-cm midline defect 4 years after hernia repair

In nine (47%) of patients who had a CT, the mesh had stretched across the defect by an average of 21.3% (range 5.7–40%). This was observed for both small and large defects—Figs. 1 and 2.

## Discussion

This study shows that 3 (9.4%) of 32 patients had a late recurrence of their hernia following laparoscopic repair of a ventral hernia using a polyester mesh. Recurrences were only observed after 5 years of follow-up. This pattern of recurrence has not been reported before and highlights the importance of long-term follow-up in this group of patients. Around 50% of recurrences for both open and laparoscopic ventral hernia are thought to happen within 2 years of operation, yet in this study none were recorded at that time point [4].

A possible explanation for hernia recurrence particularly after bridging a defect could be loss of elasticity of the mesh with herniation of the mesh into the defect. MRI studies have shown that an intraabdominal pressure of 18.6 Kpa generates a force on the abdominal wall of 28 n/cm in the transverse direction and 22 n/cm in the longitudinal direction [5]. This pressure can be generated by coughing or jumping in a healthy adult and far outstrips the force required to convert the mesh used in this study from elastic to plastic [6].

Although CT scans confirmed stretching of the polyester mesh used to bridge the defect, this was not present in all patients and in some it was minor and not clinically relevant. Degradation of the mesh over time, combined with stretching, seems a more logical explanation of the late recurrence seen in our study. A study by Riepe et al. examining the in vivo hydrolysis of polyester vascular grafts demonstrated that hydrolytic degradation of polyester reduced their bursting pressure by 31.4% at 10 years [7]. Degradation is obviously observed for all mesh products and is likely to weaken the mesh over time so that rupture force is considerably less than it was de novo [8–10].

Differential outcome for different meshes have been observed following both open and laparoscopic ventral hernia repair [2, 4, 11]. Generally lightweight meshes break and give rise to early recurrence. There is some evidence that suturing a defect rather than just bridging it reduces early recurrence [1]. However, closing the defect was not performed in this study and yet all recurrences were seen after 5 years.

One of the drawbacks of this study is the lack of long-term clinical follow-up. However, in the current climate of Covid-19 this would not be possible. An alternative is to look at recurrence through well-kept national registries. This identifies patients that undergo reoperation for their hernia and if we were to do that only 1 (3%) of the recurrences in our study would have been found. As many patients require a CT scan for one reason or another as they get older, while not perfect, this will identify the patient who is asymptomatic or does not wish a further operation for their hernia. Scotland has a national linked picture archiving and communication system (PACS) which is in effect a comprehensive imaging registry for our population.

A further drawback of this study is that the number of patients in the study was small and represented only one-third of those referred for operation over the 2-year period. However, all the operations were performed by an experienced laparoscopic surgeon with a major interest in hernia management. The unit acted as a tertiary referral centre for complex hernias with 18 of the patients having contaminated wounds or dirty wounds secondary to infected mesh or fistulae while the remainder had complex often recurrent hernias suitable for retro-muscular or component separation repair only.

## Conclusion

This study shows that late recurrence is a problem following laparoscopic ventral hernia repair with polyester mesh. The mesh loses it elasticity at a low force. This combined with degradation of polyester mesh seems the most likely cause of failure. This is unlikely to be a unique problem of polyester mesh and further long-term studies are required to better assess this operative approach to ventral hernia repair.

## Data Availability

Available if requested.
